# Feasibility analysis of combined surgery for esophageal cancer

**DOI:** 10.1186/s12957-023-02930-0

**Published:** 2023-02-10

**Authors:** Zhulin Wang, Shaowu Sun, Kaiyuan Li, Chunyao Huang, Xu Liu, Guoqing Zhang, Xiangnan Li

**Affiliations:** Department of Thoracic Surgery, First Affiliated Hospital of Zhengzhou University, Zhengzhou, 450052 Henan Province China

**Keywords:** Esophageal cancer, Combined surgery, PSM, Postoperative complications, Anastomotic leakage

## Abstract

**Background:**

As the preoperative examination of esophageal cancer has improved, the likelihood of finding diseases in other organs that require surgical treatment has also increased. The purpose of this study was to explore the feasibility of combined surgery for esophageal cancer by analyzing the occurrence of postoperative complications in patients with esophageal cancer.

**Methods:**

The clinical characteristics of 1566 patients with esophageal cancer who underwent thoracic surgery in our hospital between January 2017 and September 2022 were analyzed retrospectively. The feasibility of combined surgery for esophageal cancer was analyzed by comparing postoperative complications in patients who underwent simple esophageal cancer surgery (SEC) with those in patients who underwent combined surgery for esophageal cancer (COEC). The tendency scores of patients in the COEC and SEC groups (1:2) were matched to balance the confounding clinical factors, and the difference in postoperative complications was further analyzed. Moreover, we performed a subgroup analysis of esophagectomy combined with lung resection (ECL). In addition, the independent risk factors for postoperative Clavien–Dindo ≥ grade III complications of esophageal cancer were analyzed by multivariate logistic regression.

**Results:**

A total of 1566 patients (1147 (73.2%) males and 419 (26.8%) females), with an average age of 64.2 years, were analyzed. There was no significant difference in postoperative complications between the SEC and COEC groups according to the Clavien-Dindo classification (*P*=0.713). An analysis of the complications revealed that those in the COEC group had a higher incidence of lung consolidation than those in the SEC group (*P*=0.007). However, when we performed propensity score matching (PSM) on the SEC and COEC groups, there was still no significant difference in complications according to the Clavien–Dindo classification (*P*=0.346); furthermore, when a detailed analysis of complications was performed, there was no significant difference between the two. In subgroup analysis, after we performed PSM in ECL patients and SEC patients, we also found no significant difference in postoperative complications between patients with ECL and patients with SEC. In addition, we found that a history of diabetes (OR=1.604, *P*=0.029, 95% CI=1.049–2.454), a history of coronary heart disease (OR=1.592, *P*=0.046, 95% CI=1.008–2.515), diffusing capacity of the lungs for carbon monoxide (DLCO) (OR=0.916, *P*=0.024, 95% CI=0.849–0.988), and ALB level (OR=0.955, *P*=0.007, 95% CI=0.924–0.987) were independent factors that influenced postoperative complications in esophageal cancer patients with grade III or higher complications.

**Conclusion:**

Combined surgery for esophageal cancer does not increase the incidence of postoperative complications. In addition, a history of diabetes mellitus or coronary heart disease, carbon monoxide dispersion, and preoperative ALB level are independent risk factors for grade III or higher postoperative complications of esophageal cancer.

**Supplementary Information:**

The online version contains supplementary material available at 10.1186/s12957-023-02930-0.

## Introduction

Esophageal cancer is the eighth most common type of cancer in the world and the sixth leading cause of cancer-related death [1, 2], with a 5-year overall survival rate of approximately 20% [3–5]. Thoracoscopic surgery for esophageal cancer has become a widely recognized minimally invasive procedure [6]. However, postoperative complications of esophageal cancer still occur, and complications such as anastomotic leakage significantly increase patient mortality [7]. In addition, previous studies [8] have shown that there is a significant correlation between complications and the long-term survival of patients with esophageal cancer.

As the preoperative examination of esophageal cancer has improved, the likelihood of finding diseases in other organs that require surgical treatment has also increased. For patients with other organ diseases found at the same time, we found no relevant report recommending combined surgery or secondary surgery. Therefore, we analyzed the postoperative complications of esophageal cancer with or without other operations to explore the feasibility of combined surgery for esophageal cancer. In our study, patients with esophageal cancer were divided into patients who underwent simple esophageal cancer surgery (SEC) and patients who underwent combined surgery for esophageal cancer (COEC). The difference in postoperative complications was analyzed to investigate the feasibility of combined surgery. We also analyzed the influencing factors of serious postoperative complications of esophageal cancer (Clavien–Dindo grade ≥ III).

## Materials and methods

### Patients

This study was approved by the Ethics Committee of the First Affiliated Hospital of Zhengzhou University. The review committee did not require the completion of informed consent because of the observational and retrospective nature of the study. A total of 1566 patients with esophageal cancer were admitted to the thoracic surgery department of our hospital between January 2017 and September 2022, regardless of sex, age, height, weight, and other basic information. Clinical information included any diseases/conditions present before surgery, preoperative laboratory indicators, surgical information, and postoperative complications. Cardiopulmonary functions (forced expiratory volume in 1 s (FEV1), forced vital capacity (FVC), ejection fraction (EF), etc.) were evaluated before surgery in all patients. In combined esophageal surgery, two or more different operations are performed at the same time, one of which is for esophageal cancer. CT images were reexamined in the outpatient unit 30 days after the operation, and patients with suspected complications were reexamined and treated in the hospital.

### Combined surgery preoperative preparation and incision selection

Before surgery, the patient’s tumor and comorbidities should be evaluated based on the patient’s medical history and related examinations (for example, pulmonary function, cardiac function, chest CT, abdominal CT, neck color doppler ultrasound, head magnetic resonance, etc.). In addition, before we consider performing a combined operation on the patient, we fully evaluate the cardiopulmonary function of the patient, and only patients with a good physical condition can undergo the combined operation. The need for a combined operation was discussed by the multidisciplinary team before the operation. Additionally, in thyroid surgery, the cervical incision is changed from a standard cervical incision along the medial side of the sternocleidomastoid muscle to a transverse incision along the cervical dermatoglyphics. In combined lung surgery, the main operating port is extended to about 3 cm, but only right lung combined surgery was performed in our study. When combined with mediastinal or abdominal surgery, an incision typically made in the radical resection of esophageal cancer is preferred, and if thoracic or laparoscopic surgery cannot be completed for any reason, the operation should be changed to thoracotomy or laparotomy. In addition, when combined with pancreatic serous cystadenoma, etc., if the patient can tolerate surgery, even if preoperative multi-disciplinary team consultation considers that laparotomy is required, laparotomy should be combined, because elective secondary surgery is more difficult. However, for liver cyst fenestration and other laparoscopic operations, the original radical esophagectomy incision is selected.

### Patient selection

**Fig. 1:**
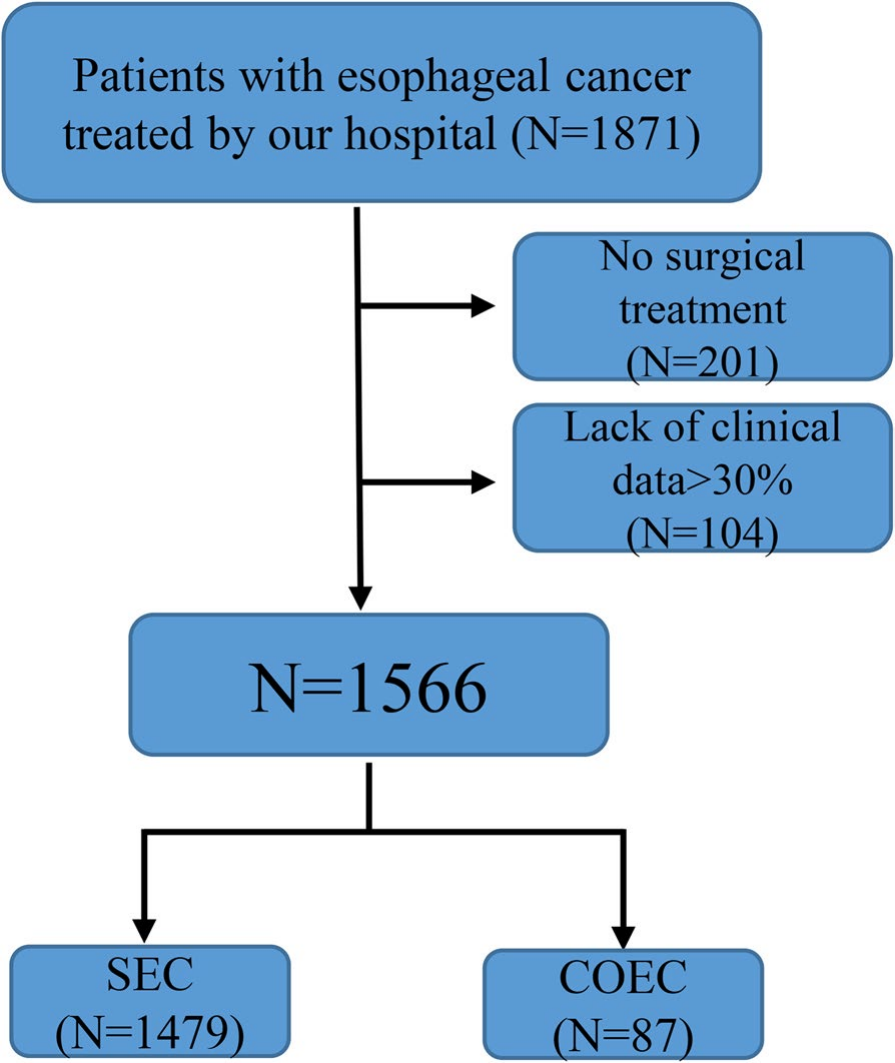
Flow chart of patient screening

The inclusion criteria were as follows: (1) patients with a pathological diagnosis of esophageal cancer (neoadjuvant therapy is accepted), (2) patients who underwent radical resection of esophageal cancer (including thoracoscopic surgery, open surgery, and conversion to thoracotomy), and (3) patients aged older than 18 years but younger than 80 years. The exclusion criteria were as follows: (1) patients with esophageal cancer who had not received surgical treatment and (2) patients with significant data missing in medical records (>30%), as identified during the screening process (Fig. 1).

### Definition and classification of postoperative complications

We used the European Perioperative Clinical Outcome (EPCO) definitions to define complications [9]. Anesthetic risk was assessed by the ASA physical status classification system [10]. Patients’ comorbidities were assessed by the Charlson comorbidity index (CCI) [11]. In addition, in-hospital death was defined as death from any cause during the period of hospitalization. Postoperative complications were graded using the Clavien–Dindo classification and the Extended Clavien–Dindo classification of surgical complications: Japanese Clinical Oncology Group postoperative complications criteria (JCOG PC criteria) [12]. The study included primary endpoints (Clavien–Dindo scores) and secondary endpoints (hospital death, anastomotic fistula, anastomotic stenosis, respiratory failure, pulmonary complications, postoperative hospital stay, etc.).

### Statistical analysis

We used IBM SPSS22.0 software (IBM SPSS Statistics, Version 22.0; IBM Corp., Armonk, NY, USA) and R language for data processing, statistical analysis, and drawing. The chi-square test or Fisher’s exact probability method was used to compare the quantitative data between groups. Propensity score matching (PSM) was performed to reduce the potential impact of selection bias and 0.2 was used as the caliper. A logistic regression model was established to calculate the propensity score based on the following covariables: age, sex, BMI, smoking history, drinking history, any diseases/conditions present before surgery, cardiopulmonary function index, neoadjuvant therapy, mode of operation, and tumor-related information. Patients in the COEC and SEC groups were matched 1:2 according to the tendency score. Moreover, we used the absolute standard mean difference (SMD) to assess the balance of the covariates after matching. In addition, we performed a subgroup analysis of esophagectomy combined with lung resection (ECL). Variables that met the criterion of *P* < 0.05 in the univariate analysis were entered into the multivariate logistic regression model, which was used to analyze the independent risk factors for postoperative complications of esophageal cancer with Clavien–Dindo grade III or above.

## Results

### Patient characteristics

**Fig. 2:**
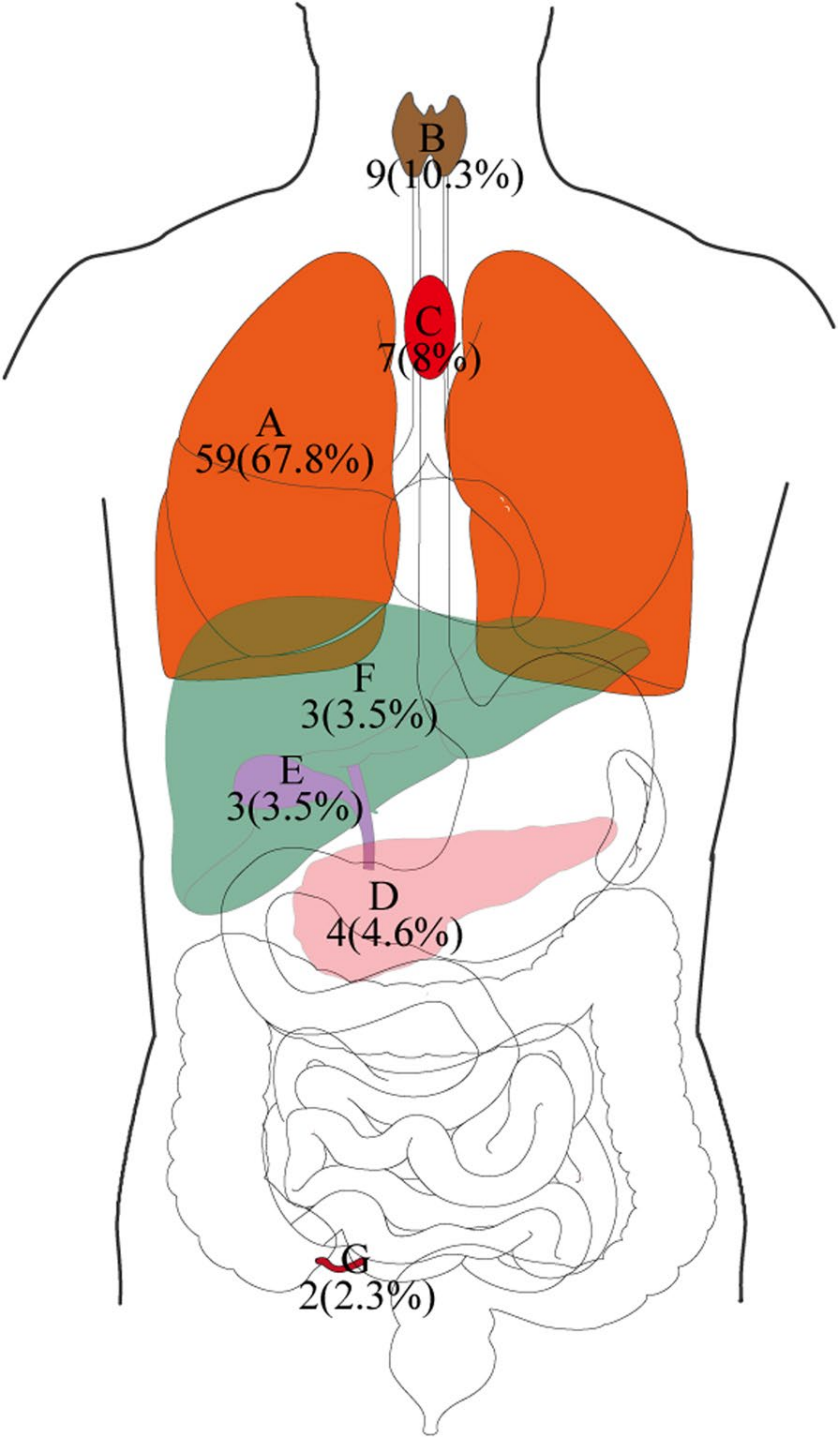
Combined surgical sites for esophageal cancer (**A** lung, **B** thyroid, **C** mediastinal, **D** pancreas, **E** gallbladder, **F** liver, **G** appendix)

**Fig. 3:**
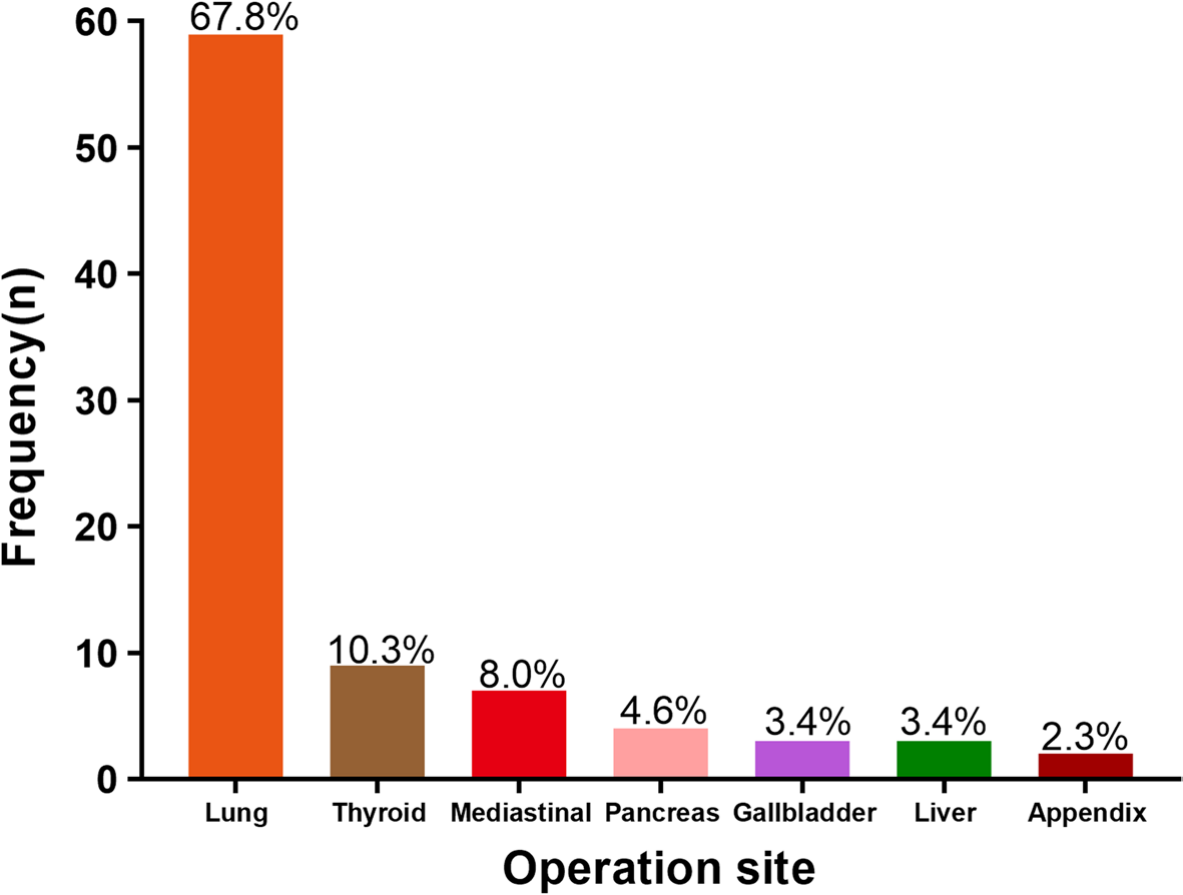
Frequency of combined surgical site for esophageal cancer represented by histogram

A total of 1566 patients with esophageal cancer (1147 males (73.2%) and 419 females (26.8%)), with an average age of 64.2 years, were included. There were 1479 (94.4%) patients who underwent simple esophageal cancer surgery (SEC) and 87 (5.6%) patients who underwent COEC (Fig. 1). The most common site of combined surgery was the lungs (59 cases), followed by the thyroid (9 cases) (Figs. 2 and 3). In addition, among the 87 patients who underwent surgery during the same period, the most common operations were wedge resection (17 cases) and bullectomy (16 cases). We listed the operation information of 87 patients in detail in Supplementary Table 1. There were 352 patients (22.5%) with a history of previous surgery, the most common of which was appendectomy. Moreover, we also listed the type of the top 10 previous surgeries (Supplementary Figure 1). In our study, 1415 (90.4%) patients had esophageal cancer, 151 patients (9.6%) had gastroesophageal junction cancer, and 368 (23.5%) patients received neoadjuvant therapy (immunity therapy, chemotherapy, and chemotherapy, etc.) before the operation. The study also included 1275 (81.4%) patients with esophageal squamous cell carcinoma and 195 (12.5%) patients with adenocarcinoma (Supplementary Table 3). The McKeown operation accounts for 86.7% of all esophageal cancer operations. The incidence of postoperative complications was 64.1%, 303 (19.3%) patients had complications above Clavien–Dindo class III, 24 patients (1.5%) died during hospitalization, and the average postoperative hospital stay was 12.04 days (Supplementary Table 4).

### Feasibility analysis of combined surgery for esophageal cancer

We analyzed the clinicopathological features and postoperative complications of patients who underwent SEC or COEC. We found a higher proportion of COEC patients with a history of alcohol consumption (*P*=0.047) and hypertension (*P*=0.004) than SEC patients. Additionally, more COEC patients received neoadjuvant therapy before surgery (*p*=0.049). Furthermore, we compared the CCI and ASA scores between the two groups and found that there was no significant difference in the CCI and ASA scores between the two groups (Supplementary Table 2). There was no significant difference in tumor characteristics (including tumor location, size, pathological type, and TNM stage) between the two groups. In addition, there was no significant difference between the two groups in terms of total length of hospital stay (*P*=0.529) or length of ICU stay (*P*=0.500). However, patients who underwent COEC required a longer operation time (*P* < 0.001) (Supplementary Table 3).

When we analyzed the postoperative complications of the patients, we found no significant difference in Clavien–Dindo classification between the two groups (*P*=0.713). Further analysis of the complications of the patients showed that the incidence of lung consolidation in the COEC group was significantly higher than that in the SEC group (*P*=0.007), and there was no significant difference in other lung complications between the two groups. In addition, there was no significant difference between the two groups in terms of the incidence of anastomotic leakage (*P*=0.464), anastomotic stenosis (*P*=0.922), cardiac complications (*P*=0.930), deep vein thrombosis (*P*=0.212), or other complications (Supplementary Table 4). The incidence of serious complications such as respiratory failure (*P*=0.788) and death during hospitalization (*P*=0.594) was not significantly different between the two groups (Supplementary Table 4).

### Propensity score matching (PSM) analysis

To reduce the effect of differences in clinical factors on the results, we performed 1:2 PSM between the COEC and SEC groups, and 87 patients in the COEC group and 174 patients in the SEC group were successfully matched. There was no significant difference in clinicopathological features between the two groups after pairing, and the SMD of each covariate was less than 0.2 (Table 1), indicating that PSM effectively balanced the confounding variables between the two groups. After PSM, we found no significant differences in postoperative complications, including anastomotic leakage (*P*=0.270), anastomotic stenosis (*P*=0.866), pulmonary complications (including pneumonia (*P*=0.689), atelectasis (*P*=0.119), pulmonary consolidation (*P*=0.455), pleural effusion (*P*=0.231)), cardiac complications (*P*=0.238), or deep vein thrombosis (*P*=0.900), between the patients in the two groups. At the same time, after propensity score matching, we found no significant difference in the total length of hospital stay (*P*=0.258) or the length of ICU stay (*P*=0.731) between the two groups (Table 2).

**Table 1:** Clinicopathological characteristics of patients after propensity score matching

Characteristic	SEC (*N*=174)	COEC (*N*=87)	*P*	SMD
**Age**	62.99±7.782	63.30±6.847	0.752	0.042
**Sex**			0.391	0.111
Male	140 (80.5%)	66 (75.9%)		
Female	34 (19.5%)	21 (24.1%)		
**BMI**	23.74±3.381	23.81±2.427	0.861	0.024
**Smoking**			0.334	0.127
No	91 (59.0%)	51 (58.6%)		
Yes	83 (41.0%)	36 (41.4%)		
**Drinking**			0.523	0.084
No	119 (68.8%)	58 (66.7%)		
Yes	54 (31.2%)	29 (33.3%)		
**Lung disease**			0.270	0.150
No	157 (90.2%)	82 (94.3%)		
Yes	17 (9.8%)	5 (5.7%)		
**Diabetes**			0.781	0.037
No	154 (88.5%)	78 (89.7%)		
Yes	20 (11.5%)	9 (10.3%)		
**Hypertension**			1.000	<0.001
No	108 (632.1%)	54 (62.1%)		
Yes	66 (37.9%)	33 (37.9%)		
**Coronary heart disease**			1.000	<0.001
No	168 (96.6%)	84 (96.6%)		
Yes	6 (3.4%)	3 (3.4%)		
**Surgical history**			0.830	0.028
No	138 (79.3%)	68 (78.2%)		
Yes	36 (20.7%)	19 (21.8%)		
**FVC**	3.63±0.750	3.58±0.717	0.614	0.067
**FEV1**	2.71±0.628	2.69±0.613	0.856	0.024
**DLCO**	7.13±1.773	7.18±1.719	0.850	0.025
**EF**	63.44±2.307	63.39±2.384	0.866	0.022
**ALB**	40.37±3.515	39.99±4.200	0.450	0.096
**Neoadjuvant therapy**			1.000	<0.001
No	118 (67.8%)	59 (67.8%)		
Yes	56 (32.2%)	28 (32.2%)		
**Surgical procedures**			0.898	0.017
McKeown	151 (86.8%)	75 (86.2%)		
Others^a^	23 (13.2%)	12 (13.8%)		
**Tumor location**			0.168	0.102
Upper	33 (19.0%)	10 (11.5%)		
Middle	65 (37.4%)	35 (40.2%)		
Lower	65 (37.4%)	40 (46.0%)		
GEJ	11 (6.3%)	2 (2.3%)		
**Tumor size (cm)**	3.16±1.443	3.16±1.532	0.988	0.002
**Histological type**			0.738	0.040
Squamous	154 (88.5%)	75 (86.2%)		
Adenocarcinoma	15 (8.6%)	10 (11.5%)		
Other	5 (2.9%)	2 (2.3%)		
**TNM stage**			0.825	0.075
1	65 (37.4%)	30 (34.5%)		
2	62 (35.6%)	32 (36.8%)		
3	44 (25.3%)	22 (25.3%)		
4	3 (1.7%)	3 (3.4%)		

*BMI* body mass index, *FVC* forced vital capacity, *FEV1* forced expiratory volume in one second, *DLCO* diffusing capacity of the lung for carbon monoxide, *EF* ejection fraction

Others^a^including Sweet esophagectomy, Ivor-Lewis esophagectomy, right thoracotomy with cervical anastomosis, mediastinoscopy-assisted esophagectomy, *GEJ* gastro-oesophageal junction cancers

**Table 2:** Comparison of complications in patients after propensity score matching

Characteristics	SEC (*N*=174)	COEC (*N*=87)	*P*
**Clavien-Dindo**			0.346
1–2	73 (42.0%)	37 (42.5%)	
3	37 (21.3%)	11 (12.6%)	
4–5	7 (4.0%)	4 (4.6%)	
**Anastomotic stenosis**			0.866
No	161 (92.5%)	81 (93.1%)	
Yes	13 (7.5%)	6 (6.9%)	
**Anastomotic leakage**			0.270
No	157 (90.2%)	82 (94.3%)	
Yes	17 (9.8%)	5 (5.7%)	
**Pneumonia**			0.689
No	128 (73.6%)	66 (75.9%)	
Yes	46 (26.4%)	21 (24.1%)	
**Atelectasis**			0.119
No	129 (74.1%)	72 (82.8%)	
Yes	45 (25.9%)	15 (17.2%)	
**Pulmonary consolidation**			0.455
No	139 (19.9%)	66 (75.9%)	
Yes	35 (20.1%)	21 (24.1%)	
**Pleural effusion**			0.231
No	141 (81.5%)	76 (87.4%)	
Yes	32 (18.5%)	11 (12.6%)	
**Cardiac complication**			0.238
No	142 (81.6%)	75 (86.2%)	
Yes	32 (18.4%)	12 (13.8%)	
**DVT**			0.900
No	149 (85.6%)	80 (92.0%)	
Yes	25 (14.4%)	7 (8.0%)	
**Operation time**	311.55±53.730	340.77±32.191	<0.001^*^
**Intraoperative infusion**	3371.38±634.549	3313.22±537.677	0.464
**Total in-hospital stay**	20.51±6.321	21.31±4.246	0.258
**Length of postoperative hospital stay**	11.96±4.231	12.32±2.394	0.460
**ICU stay**	3.31±1.888 (*n*=13)	3.00±0.707 (*n*=5)	0.731

*DVT* deep vein thrombosis

### Subgroup analysis of esophagectomy combined with lung resection

We analyzed the subgroup of patients who underwent esophagectomy combined with lung resection. In our study, there were 59 subgroup patients with esophagectomy combined with lung resection, 48 of them were male (81.4%), the average age was 62.86 years, and the average operation time was 337.83 min (Supplementary Table 5). In the patients who underwent ECL, lung surgical procedures included wedge resection (17 cases), bullectomy (16 cases), lobectomy (14 cases), and segmentectomy (12 cases) (Supplementary Table 1). In propensity score matching, using a 1:2 ratio, we matched ECL patients with SEC patients; a total of 118 SEC patients were successfully matched. There was no significant difference in the clinicopathological characteristics of the two groups after pairing, and the SMDs of each covariate were less than 0.2 (Supplementary Table 5), indicating that the PSM effectively balanced the confounding variables between the two groups. There was no significant difference in the Clavien-Dindo classification (*P*=0.628) of postoperative complications between the two groups after PSM. Moreover, we performed a detailed analysis of complications, including anastomotic leakage (*P*=0.154), anastomotic stenosis (*P*=0.173), pulmonary complications (including pneumonia (*P*=0.907), atelectasis (*P*=0.790), pulmonary consolidation (*P*=0.566), pleural effusion (*P*=0.352)), cardiac complications (*P*=0.874), and deep vein thrombosis (*P*=0.506) and found no significant difference between the two groups. In the subgroup analysis of esophagectomy combined with lung resection, we also found no significant difference in the total length of hospital stay (*P*=0.799) or the length of ICU stay (*P*=0.618) between the two groups (Table 3).

**Table 3:** Comparison of complications between patients who underwent esophagectomy combined with lung resection and patients with esophagectomy alone after PSM

Characteristic	SEC (*N*=118)	ECL (*N*=59)	*P*
**Clavien-Dindo**			0.628
1–2	56 (47.5%)	24 (40.7%)	
3	13 (11.0%)	9 (15.3%)	
4–5	7 (5.9%)	2 (3.4%)	
**Anastomotic stenosis**			0.173
No	115 (97.5%)	55 (93.2%)	
Yes	3 (2.5%)	4 (6.8%)	
**Anastomotic leakage**			0.154
No	107 (90.7%)	57 (96.6%)	
Yes	11 (9.3%)	2 (3.4%)	
**Pneumonia**			0.907
No	83 (70.3%)	42 (71.2%)	
Yes	35 (29.7%)	17 (28.8%)	
**Atelectasis**			0.790
No	94 (79.7%)	48 (81.4%)	
Yes	24 (20.3%)	11 (18.6%)	
**Pulmonary consolidation**			0.566
No	100 (84.7%)	48 (81.4)	
Yes	18 (15.3%)	11 (18.6%)	
**Pleural effusion**			0.352
No	104 (88.1%)	49 (83.1%)	
Yes	14 (11.9)	10 (16.9%)	
**Cardiac complication**			0.874
No	103 (87.3%)	51 (86.4%)	
Yes	15 (12.7%)	8 (13.6%)	
**DVT**			0.526
No	109 (92.4%)	56 (94.9%)	
Yes	9 (7.6%)	3 (5.1%)	
**Operation time**	311.03±43.454	337.83±16.695	<0.001
**Intraoperative infusion**	3324.32±537.826	3295.76±473.276	0.730
**Total in-hospital stay**	21.09±8.014	20.78±4.259	0.799
**Length of postoperative hospital stay**	12.17±4.024	12.37±2.243	0.719
**ICU stay**	3.75±2.375 (*n*=8)	3.00±1.000 (*n*=3)	0.618

*DVT* deep vein thrombosis

### Univariate and multivariate analyses of complications of Clavien–Dindo grade III and above

We performed univariate and multivariate analyses on grade III or higher postoperative complications in patients with esophageal cancer. In the univariate analysis, we found that a history of diabetes (OR=1.702, *P*=0.011, 95% CI=1.127–2.571), a history of coronary heart disease (OR=1.741, *P*=0.014, 95% CI=1.117–2.712), diffusing capacity of the lungs for carbon monoxide (DLCO) (OR=0.894, *P*=0.003, 95% CI=0.830–0.964), ALB (OR=0.950, *P*=0.002, 95% CI=0.919–0.981), gastro-esophageal junction cancers (OR=0.513, *P*=0.023, 95% CI=0.288–0.914), esophageal adenocarcinoma (OR=0.637, *P*=0.039, 95% CI=0.415–0.977), operation time (OR=1.003, *P*=0.009, 95% CI=1.001–1.006), and intraoperative infusion volume (OR=1.001, P=0.005, 95% CI=1.000–1.001) were potential factors influencing the occurrence of grade III or higher postoperative complications in patients with esophageal cancer (Table 4). In the multivariate analysis, we found that a history of diabetes (OR=1.604, *P*=0.029, 95% CI=1.049–2.454), a history of coronary heart disease (OR=1.592, *P*=0.046, 95% CI=1.008–2.515), DLCO (OR=0.916, *P*=0.024, 95% CI=0.849–0.988), and ALB level (OR=0.955, *P*=0.007, 95% CI=0.924–0.987) independently influenced the incidence of grade III or higher postoperative complications in patients with esophageal cancer (Table 4).

**Table 4:** Univariate and multivariate analyses of Clavien–Dindo grade III and above complications

Characteristic	Univariate			Multivariable		
	OR	95% CI	*P* value	OR	95% CI	*P* value
**Operation**						
SEC	1					
COEC	0.862	0.487–1.525	0.609			
**Age**	1.008	0.992–1.025	0.312			
**Sex**	1.129	0.855–1.492	0.392			
**BMI**	0.973	0.937–1.010	0.146			
**Smoking**	1.062	0.821–1.373	0.646			
**Drinking**	1.161	0.873–1.545	0.305			
**Lung disease**	1.073	0.653–1.762	0.782			
**Diabetes**	1.702	1.127–2.571	0.011^*^	1.604	1.049–2.454	0.029^*^
**Hypertension**	0.978	0.731–1.307	0.878			
**Coronary heart disease**	1.741	1.117–2.712	0.014^*^	1.592	1.008–2.515	0.046^*^
**FVC**	0.862	0.735–1.010	0.067			
**FEV1**	0.854	0.703–1.037	0.111			
**DLCO**	0.894	0.830–0.964	0.003^*^	0.916	0.849–0.988	0.024^*^
**EF**	0.970	0.917–1.026	0.284			
**WBC**	0.998	0.940–1.060	0.956			
**ALB**	0.950	0.919–0.981	0.002^*^	0.955	0.924–0.987	0.007^*^
**N**	0.994	0.948–1.043	0.810			
**LN**	0.938	0.813–1.082	0.381			
**M**	0.961	0.793–1.165	0.685			
**Tumor location**						
Upper	1					
Middle	0.968	0.660–1.420	0.866			
Lower	0.809	0.556–1.176	0.266			
GEJ	0.513	0.288–0.914	0.023^*^	-	-	-
**Histological type**						
Squamous	1					
Adenocarcinoma	0.637	0.415–0.977	0.039^*^	-	-	-
Other	0.977	0.581–1.645	0.931			
**Tumor size (cm)**	0.988	0.908–1.073	0.768			
**TNM stage**						
1						
2	0.911	0.671–1.237	0.550			
3	0.914	0.663–1.260	0.583			
4	0.791	0.419–1.491	0.468			
**Operation time**	1.003	1.001–1.006	0.009^*^	-	-	-
**Intraoperative infusion**	1.000	1.000–1.001	0.005^*^	-	-	-
**Surgical procedures**	0.695	0.464–1.040	0.077			

## Discussion

In this study, we analyzed postoperative complications in 1566 patients who underwent esophageal cancer surgery. In the 87 patients who underwent combined surgery, we found no significant difference in the incidence of postoperative complications compared with patients who underwent surgery for esophageal cancer alone. Even after strict PSM, there was no significant difference in postoperative complications between the two groups. In our subgroup analysis of patients with esophagectomy combined with lung resection, we found that there was still no significant difference in complications between the patients who underwent esophagectomy combined with lung resection and the patients who underwent esophagectomy alone. This suggests that combined surgery for esophageal cancer is feasible because it only marginally increases the operation time. In addition, we identified the factors that independently influenced grade III and above postoperative complications in patients with esophageal cancer, which included a history of diabetes, a history of coronary heart disease, carbon monoxide diffusing capacity, and ALB. Our results indicate that combined surgery does not impact the occurrence of grade III or higher postoperative complications in patients with esophageal cancer.

Esophageal cancer is an extremely serious disease that is difficult to cure, owing to the frequent occurrence of various complications after esophagectomy. A 14-country study by the Esophageal Cancer Complications Group (ECCG) showed [13] that the overall complication rate after esophageal cancer surgery was 59%. In our study, the overall rate of postoperative complications in patients with esophageal cancer was 64.1%; the rate of Clavien–Dindo grade III or higher complications was 19.3%, and the in-hospital mortality rate was 1.5%. Some previous studies [14–17] have shown that complications after esophageal cancer surgery can lead to poor prognosis in patients with esophageal cancer. In addition, both the study by Ayako [18] et al. and the study by Eisuke [8] et al. found that postoperative pneumonia after esophageal cancer was an independent risk factor for poor prognosis. In addition, in the study of Li [19] et al., 26 patients with both esophageal and gastric cancer had no significant difference in median OS compared with patients with esophageal cancer alone. Kato [20] et al. found no significant difference in mortality, intraoperative bleeding, or postoperative complications between patients who underwent combined esophageal and lung surgery versus those who underwent esophageal cancer surgery alone, consistent with our findings.

In our study, we found that a history of diabetes mellitus, a history of coronary heart disease, carbon monoxide diffusing capacity, and ALB were factors that independently influenced the occurrence of grade III or above postoperative complications in patients with esophageal cancer. Daniel [21] et al. analyzed 2315 postoperative patients with esophageal cancer and found that diabetes was an important predictor of postoperative complications. Lovisa [22] et al. found that comorbid heart disease increased the risk of postoperative complications in patients with esophageal cancer. The study by Goense [23] et al. showed that DLCO is an independent predictor of major complications after esophagectomy for cancer. ALB is used to reflect the nutritional status of patients and the ongoing systemic inflammatory response [24]. Therefore, patients with low ALB may have a lower tolerance for surgery and are more likely to develop postoperative complications. In addition, Lv [25] et al. found that the ratio of neutrophils to ALB was a prognostic indicator for esophageal squamous cell carcinoma. Patients with preoperative diabetes, coronary heart disease, and lower DLCO and ALB levels had an increased risk of postoperative complications of grade 3 or higher, leading to a poor prognosis. Therefore, regardless of whether patients undergo simple esophageal surgery or combined surgery, it is necessary to strengthen the perioperative management, especially the control of the underlying disease, the improvement of lung function, and nutritional support.

Although combined surgery for esophageal cancer prolongs the operation time, it does not increase the incidence of postoperative complications and mortality. Combined esophageal surgery can benefit the patient by reducing the number of hospitalizations and the number of operations. When esophageal surgery is combined with nonthoracic surgery, attention should be given to the coordination of multidisciplinary surgery.

Our study is the first to provide a detailed analysis of the feasibility of combined surgery for esophageal cancer. There was no significant difference in the incidence of postoperative complications and mortality between the two groups.

### Limitation

This study is a single-center study, so the conclusions may be biased. The sequence of the combined operations, whether lung resection or freeing of the esophagus was performed first in esophagectomy combined with lung resection, was not included in the study. The impact of omittance on the results needs follow-up research. In the subgroup analysis, due to the sample size, we only analyzed the esophagectomy combined with lung resection subgroups. In follow-up studies, it is necessary to increase the sample size and include resections of other sites (such as cholecystectomy, liver resection, etc.) to further prove the feasibility of combined surgery for esophageal cancer. In addition, whether combined surgery for esophageal cancer has an impact on the long-term prognosis of patients with esophageal cancer needs further study.

## Conclusion

Esophageal cancer combined with other surgeries does not increase the incidence of postoperative complications. In addition, a history of diabetes, a history of coronary heart disease, DLCO, and preoperative ALB levels are factors that independently influence the occurrence of grade III or higher complications after esophageal cancer surgery.

## Supplementary Information

**Additional file 1: Supplementary Table 1.** Information of 87 patients undergoing combined surgery. **Supplementary Table 2.** Baseline characteristics of the patients. **Supplementary Table 3.** Information about the patient's surgery. **Supplementary Table 4.** Postoperative complications in patients with esophageal cancer. **Supplementary Table 5.** Clinicopathological characteristics of the subgroup of patients undergoing esophagectomy combined with lung resection after PSM.

**Additional file 2: Supplementary Figure 1.** Patients with previous surgery history, top 10 surgical method.

## Data Availability

The datasets used and/or analyzed during the current study are available from the corresponding author on reasonable request.

## Abbreviations

SEC: Simple esophageal cancer surgery
COEC: Combined surgery for esophageal cancer
ECL: Esophagectomy combined with lung resection
OR: Odds ratio
CI: Confidence interval
BMI: Body mass index
FVC: Forced vital capacity
FEV1: Forced expiratory volume in one second
DLCO: Diffusing capacity of the lung for carbon monoxide
EF: Ejection fraction
GEJ: Gastro-esophageal junction cancers
PSM: Propensity score matching
SMD: Standard mean difference
ECCG: Esophageal Cancer Complications Group
JCOG: Japanese Clinical Oncology Group
EPCO: European Perioperative Clinical Outcome
