# Supplementary material for: Feasibility analysis of combined surgery for esophageal cancer
Source: World J Surg Oncol. 2023 Feb 10;21:41. doi: 10.1186/s12957-023-02930-0 (PMC9912580; doi:10.1186/s12957-023-02930-0)
Supplement: Supplementary file 1 — Additional file 1: Supplementary Table 1. Information of 87 patients undergoing combined surgery. Supplementary Table 2. Baseline characteristics of the patients. Supplementary Table 3. Information about the patient's surgery. Supplementary Table 4. Postoperative complications in patients with esophageal cancer. Supplementary Table 5. Clinicopathological characteristics of the subgroup of patients undergoing esophagectomy combined with lung resection after PSM. [file 12957_2023_2930_MOESM1_ESM.docx]

Supplementary Table 1. Information of 87 patients who underwent combined surgery

| ID | Sex | Age | Tumor location | Histological types of ESCA | TNM stage | Comorbidities | Combined Surgical Names | Surgical history |
| --- | --- | --- | --- | --- | --- | --- | --- | --- |
| 1 | female | 70 | Lower | Squamous | Stage Ⅱ | Thyroid cancer | Thyroidectomy |  |
| 2 | male | 63 | Middle | Squamous | Stage Ⅰ | Pneumatocele | Right upper lobe bullectomy | Appendectomy |
| 3 | male | 61 | Lower | Squamous | Stage Ⅰ | Lung adenocarcinoma | Right upper lobectomy |  |
| 4 | female | 68 | Lower | Squamous | Stage Ⅱ | Minimally invasive adenocarcinoma | Right middle lobe wedge pneumonectomy |  |
| 5 | male | 68 | Lower | Squamous | Stage Ⅲ | Thyroid cancer | Thyroidectomy |  |
| 6 | male | 70 | Middle | Squamous | Stage Ⅱ | Pneumatocele | Right upper lobe bullectomy | Appendectomy |
| 7 | male | 55 | Lower | Squamous | Stage Ⅰ | Lung adenocarcinoma | Right middle lobectomy |  |
| 8 | male | 53 | Lower | Squamous | Stage Ⅲ | Pneumatocele | Right upper lobe bullectomy |  |
| 9 | male | 63 | Middle | Squamous | Stage Ⅲ | Pneumatocele | Right upper lobe bullectomy |  |
| 10 | male | 58 | Lower | Squamous | Stage Ⅰ | Lung adenocarcinoma | Right upper lobectomy |  |
| 11 | male | 58 | GEJ | Adenocarcinoma | Stage Ⅱ | Lung adenocarcinoma | Right lower lobectomy |  |
| 12 | female | 68 | Lower | Adenocarcinoma | Stage Ⅱ | Serous cystadenomas | Central pancreatectomy |  |
| 13 | male | 68 | Lower | Squamous | Stage Ⅲ | Bronchiectasis | Right middle lobectomy |  |
| 14 | female | 72 | Lower | Squamous | Stage Ⅰ | Minimally invasive adenocarcinoma | Right upper lobe wedge pneumonectomy |  |
| 15 | male | 78 | Lower | Squamous | Stage Ⅱ | Pneumatocele | Right upper lobe bullectomy |  |
| 16 | female | 71 | Middle | Squamous | Stage Ⅲ | Minimally invasive adenocarcinoma | Right middle lobe wedge pneumonectomy |  |
| 17 | male | 57 | Lower | Squamous | Stage Ⅰ | Minimally invasive adenocarcinoma | Right upper lobe segmentectomy (S1) |  |
| 18 | male | 64 | Middle | Squamous | Stage Ⅱ | Thymoma | Mediastinal tumor resection |  |
| 19 | female | 66 | Middle | Squamous | Stage Ⅲ | Serous cystadenomas | Central pancreatectomy |  |
| 20 | female | 67 | Upper | Squamous | Stage Ⅰ | Gallstone Disease | Cholecystectomy |  |
| 21 | male | 63 | Upper | Squamous | Stage Ⅰ | Pneumatocele | Right middle lobe bullectomy |  |
| 22 | male | 55 | Middle | Squamous | Stage Ⅱ | Pneumatocele | Right upper lobe bullectomy | Fracture surgery |
| 23 | male | 59 | Middle | Squamous | Stage Ⅱ | Pneumatocele | Right upper lobe bullectomy |  |
| 24 | male | 58 | Middle | Squamous | Stage Ⅱ | Lung adenocarcinoma | Right upper lobe segmentectomy (S3) |  |
| 25 | male | 59 | Middle | Squamous | Stage Ⅲ | Gallstone Disease | Cholecystectomy |  |
| 26 | male | 65 | Lower | Adenocarcinoma | Stage Ⅳ | Thymic carcinomas | Mediastinal tumor resection |  |
| 27 | male | 55 | Lower | Squamous | Stage Ⅱ | Lung adenocarcinoma | Right lower lobe segmentectomy(S6) |  |
| 28 | male | 64 | Lower | Squamous | Stage Ⅰ | Liver cyst | Cyst fenestration |  |
| 29 | male | 72 | Lower | Squamous | Stage Ⅰ | Lung adenocarcinoma | Right middle lobectomy |  |
| 30 | male | 53 | Middle | Squamous | Stage Ⅰ | Lung adenocarcinoma | Right lower lobectomy | Bullectomy |
| 31 | male | 58 | Lower | Squamous | Stage Ⅰ | Acute appendicitis | Appendectomy | Fracture surgery |
| 32 | female | 73 | Middle | Squamous | Stage Ⅰ | Thyroid cancer | Thyroidectomy |  |
| 33 | male | 49 | Lower | Adenocarcinoma | Stage Ⅲ | Pancreatic cyst | Distal pancreatectomy |  |
| 34 | male | 67 | Middle | Squamous | Stage Ⅱ | Thyroid cancer | Thyroidectomy |  |
| 35 | male | 71 | Middle | Squamous | Stage Ⅱ | Thymoma | Mediastinal tumor resection |  |
| 36 | male | 62 | Lower | Squamous | Stage Ⅰ | Thyroid cancer | Thyroidectomy |  |
| 37 | male | 59 | Upper | Squamous | Stage Ⅰ | Liver cyst | Cyst fenestration |  |
| 38 | male | 60 | Middle | Small cell carcinoma | Stage Ⅱ | Pneumatocele | Right middle lobe bullectomy |  |
| 39 | male | 68 | Lower | Squamous | Stage Ⅱ | Liver cyst | Cyst fenestration |  |
| 40 | male | 54 | Lower | Squamous | Stage Ⅱ | Minimally invasive adenocarcinoma | Right middle lobe segmentectomy(S6) |  |
| 41 | male | 53 | Upper | Adenocarcinoma | Stage Ⅰ | Thymoma | Mediastinal tumor resection |  |
| 42 | male | 59 | Middle | Squamous | Stage Ⅱ | Thyroid cancer | Thyroidectomy |  |
| 43 | female | 61 | Lower | Squamous | Stage Ⅳ | Minimally invasive adenocarcinoma | Right lower lobe segmentectomy(S6) |  |
| 44 | female | 76 | Upper | Squamous | Stage Ⅲ | Adenocarcinoma in situ | Right lower lobe segmentectomy(S9) | Myomectomy |
| 45 | male | 54 | Lower | Squamous | Stage Ⅲ | Thymoma | Mediastinal tumor resection |  |
| 46 | female | 75 | Middle | Squamous | Stage Ⅱ | Minimally invasive adenocarcinoma | Right upper lobe segmentectomy (S1) |  |
| 47 | male | 73 | Lower | Squamous | Stage Ⅱ | Adenocarcinoma in situ | Right middle lobe segmentectomy(S5) | Cholecystectomy |
| 48 | male | 69 | Middle | Squamous | Stage Ⅱ | Pneumatocele | Right upper lobe bullectomy |  |
| 49 | female | 64 | Middle | Squamous | Stage Ⅱ | Adenocarcinoma in situ | Right upper lobe segmentectomy (S2) |  |
| 50 | male | 62 | Lower | Squamous | Stage Ⅰ | Lung adenocarcinoma | Right upper lobe segmentectomy (S1) |  |
| 51 | male | 62 | Upper | Squamous | Stage Ⅱ | Pneumatocele | Right upper lobe bullectomy |  |
| 52 | male | 59 | Middle | Squamous | Stage Ⅲ | Pneumatocele | Right middle lobe bullectomy | Appendectomy |
| 53 | male | 60 | Middle | Squamous | Stage Ⅲ | Pneumatocele | Right upper lobe bullectomy |  |
| 54 | female | 55 | Middle | Squamous | Stage Ⅰ | Appendix abscess | Appendectomy |  |
| 55 | female | 45 | Middle | Adenocarcinoma | Stage Ⅱ | Minimally invasive adenocarcinoma | Right upper lobe wedge pneumonectomy | Ovariectomy |
| 56 | female | 70 | Middle | Squamous | Stage Ⅲ | Thyroid cancer | Thyroidectomy |  |
| 57 | male | 67 | Lower | Squamous | Stage Ⅰ | Pneumatocele | Right upper lobe bullectomy |  |
| 58 | male | 65 | Middle | Squamous | Stage Ⅲ | Thyroid cancer | Thyroidectomy | Fracture surgery |
| 59 | female | 63 | Lower | Squamous | Stage Ⅱ | Minimally invasive adenocarcinoma | Right middle lobe wedge pneumonectomy | Myomectomy |
| 60 | male | 66 | Middle | Squamous | Stage Ⅰ | Adenocarcinoma in situ | Right lower lobe wedge pneumonectomy | Cholecystectomy |
| 61 | male | 53 | Upper | Squamous | Stage Ⅰ | Adenocarcinoma in situ | Right upper lobe wedge pneumonectomy |  |
| 62 | male | 57 | Lower | Squamous | Stage Ⅲ | Adenocarcinoma in situ | Right lower lobe wedge pneumonectomy |  |
| 63 | male | 62 | Lower | Squamous | Stage Ⅰ | Bronchiectasis | Right middle lobectomy |  |
| 64 | male | 54 | Upper | Squamous | Stage Ⅱ | Bronchiectasis | Right lower lobectomy |  |
| 65 | male | 61 | Upper | Squamous | Stage Ⅱ | Adenocarcinoma in situ | Right middle lobe wedge pneumonectomy |  |
| 66 | male | 47 | Lower | Squamous | Stage Ⅰ | Pneumatocele | Right middle lobe bullectomy | Bullectomy |
| 67 | male | 66 | Lower | Squamous | Stage Ⅰ | Adenocarcinoma in situ | Right lower lobe wedge pneumonectomy |  |
| 68 | female | 71 | Upper | Squamous | Stage Ⅱ | Minimally invasive adenocarcinoma | Right middle lobe wedge pneumonectomy |  |
| 69 | male | 69 | Middle | Squamous | Stage Ⅰ | Minimally invasive adenocarcinoma | Right lower lobe wedge pneumonectomy |  |
| 70 | female | 67 | Middle | Squamous | Stage Ⅲ | Minimally invasive adenocarcinoma | Right lower lobe wedge pneumonectomy |  |
| 71 | male | 66 | Middle | Squamous | Stage Ⅲ | Lung adenocarcinoma | Right upper lobectomy |  |
| 72 | female | 61 | Middle | Squamous | Stage Ⅰ | Thymic carcinomas | Mediastinal tumor resection |  |
| 73 | male | 62 | Middle | Squamous | Stage Ⅱ | Lung adenocarcinoma | Right upper lobectomy |  |
| 74 | male | 74 | Lower | Squamous | Stage Ⅱ | Gallstone Disease | Cholecystectomy |  |
| 75 | male | 62 | Lower | Squamous | Stage Ⅲ | Lung adenocarcinoma | Right middle lobectomy | Nasal polypectomy |
| 76 | male | 58 | Middle | Squamous | Stage Ⅰ | Minimally invasive adenocarcinoma | Right upper lobe wedge pneumonectomy |  |
| 77 | male | 69 | Lower | Adenocarcinoma | Stage Ⅲ | Pancreatic cyst | Distal pancreatectomy | Appendectomy |
| 78 | female | 68 | Middle | Adenocarcinoma | Stage Ⅰ | Thymoma | Mediastinal tumor resection |  |
| 79 | male | 68 | Lower | Squamous | Stage Ⅲ | Lung adenocarcinoma | Right lower lobe segmentectomy(S6) | Cholecystectomy |
| 80 | male | 77 | GEJ | Adenocarcinoma | Stage Ⅳ | Lung adenocarcinoma | Right upper lobectomy |  |
| 81 | male | 64 | Lower | Adenosquamous cell carcinoma | Stage Ⅰ | Adenocarcinoma in situ | Right upper lobe segmentectomy (S1) | Fracture surgery |
| 82 | male | 62 | Middle | Squamous | Stage Ⅲ | Adenocarcinoma in situ | Right upper lobe wedge pneumonectomy |  |
| 83 | male | 62 | Lower | Squamous | Stage Ⅲ | Lung adenocarcinoma | Right middle lobectomy | Bullectomy |
| 84 | female | 72 | Lower | Squamous | Stage Ⅱ | Thyroid cancer | Thyroidectomy | Myomectomy |
| 85 | male | 64 | Lower | Squamous | Stage Ⅱ | Pneumatocele | Right upper lobe bullectomy |  |
| 86 | male | 65 | Middle | Squamous | Stage Ⅱ | Minimally invasive adenocarcinoma | Right upper lobe wedge pneumonectomy |  |
| 87 | male | 66 | Lower | Adenocarcinoma | Stage Ⅰ | Adenocarcinoma in situ | Right upper lobe wedge pneumonectomy |  |

Supplementary Table 2. Baseline characteristics of the patients

| Characteristics | SEC  (N=1479) | COEC  (N=87) | P |
| --- | --- | --- | --- |
| **Age** | 64.25±7.754 | 63.30±6.847 | 0.263 |
| **Sex** |  |  | 0.570 |
| Male | 1081(73.1%) | 66(75.9%) |  |
| Female | 398(26.9%) | 21(24.1%) |  |
| **BMI** | 23.90±3.480 | 23.81±2.427 | 0.722 |
| **Smoking** |  |  | 0.520 |
| No | 918(62.1%) | 51(58.6%) |  |
| Yes | 561(37.9%) | 36(41.4%) |  |
| **Drinking** |  |  | 0.047^*^ |
| No | 1125(76.1%) | 58(66.7%) |  |
| Yes | 354(23.9%) | 29(33.3%) |  |
| **Lung disease** |  |  | 0.748 |
| No | 1381(93.4%) | 82(94.3%) |  |
| Yes | 98(6.6%) | 5(5.7%) |  |
| **Diabetes** |  |  | 0.403 |
| No | 1363(92.2%) | 78(89.7%) |  |
| Yes | 116(7.8%) | 9(10.3%) |  |
| **Hypertension** |  |  | 0.004^*^ |
| No | 1119(75.7%) | 54(62.1%) |  |
| Yes | 360(24.3%) | 33(37.9%) |  |
| **Coronary heart disease** |  |  | 0.211 |
| No | 1377(93.1%) | 84(96.6%) |  |
| Yes | 102(6.9%) | 3(3.4%) |  |
| **Surgical history** |  |  | 0.883 |
| No | 1146(77.5%) | 68(78.2%) |  |
| Yes | 333(22.5%) | 19(21.8%) |  |
| **CCI** |  |  | 0.190 |
| 0 | 966(65.3%) | 48(55.2%) |  |
| 1 | 299(20.2%) | 20(23.0%) |  |
| 2 | 162(11.0%) | 15(17.2%) |  |
| ≥3 | 52(3.5%) | 4(4.6%) |  |
| **ASA classification** |  |  | 0.198 |
| I/II | 1158(78.3%) | 63(72.4%) |  |
| III/IV | 321(21.7%) | 24(27.5%) |  |
| **FVC** | 3.56±1.311 | 3.58±0.717 | 0.864 |
| **FEV1** | 2.63±0.649 | 2.69±0.613 | 0.420 |
| **DLCO** | 7.08±1.714 | 7.18±1.719 | 0.626 |
| **EF** | 63.32±2.192 | 63.39±2.384 | 0.295 |
| **Neoadjuvant therapy** |  |  | 0.049^*^ |
| No | 1139(77.1%) | 59(67.8%) |  |
| Yes | 340(22.9%) | 28(32.2%) |  |

BMI, body mass index; FVC, forced vital capacity; FEV1, forced expiratory volume in one second; CCI, Charlson Comorbidity Index; ASA, American Society of Anesthesiologists; DLCO, diffusing capacity of the lung for carbon monoxide; EF, [ejection](javascript:;) [fraction](javascript:;).

| Characteristic | SEC  (N=1479) | COEC  (N=87) | P |
| --- | --- | --- | --- |
| **Surgical procedures** |  |  | 0.900 |
| McKeown | 1282(86.7%) | 75(86.2%) |  |
| Others* | 197(13.3%) | 12(13.8%) |  |
| **Tumor location** |  |  | 0.066 |
| Upper | 209(14.1%) | 10(11.5%) |  |
| Middle | 480(32.5%) | 35(40.2%) |  |
| Lower | 641(43.3%) | 40(46.0%) |  |
| GEJ | 149(10.1%) | 2(2.3%) |  |
| **Tumor size(cm)** | 3.21±1.511 | 3.16±1.532 | 0.750 |
| **Histological type** |  |  | 0.282 |
| Squamous | 1200(81.1%) | 75(86.2%) |  |
| Adenocarcinoma | 185(12.5%) | 10(11.5%) |  |
| Other | 94(6.4%) | 2(2.3%) |  |
| **T stage** |  |  | 0.725 |
| 1 | 569(38.5%) | 31(35.6%) |  |
| 2 | 347(23.5%) | 23(26.4%) |  |
| 3 | 549(37.1%) | 33(37.9%) |  |
| 4 | 14(0.9%) | 0(0.0%) |  |
| **N stage** |  |  | 0.542 |
| 0 | 900(60.9%) | 59(67.8%) |  |
| 1 | 322(21.8%) | 14(16.1%) |  |
| 2 | 188(12.7%) | 11(12.6%) |  |
| 3 | 69(4.7%) | 3(3.4%) |  |
| **TNM stage** |  |  | 0.786 |
| 1 | 469(31.7%) | 30(34.5%) |  |
| 2 | 511(34.6%) | 32(36.8%) |  |
| 3 | 425(28.7%) | 22(25.3%) |  |
| 4 | 74(5.0%) | 3(3.4%) |  |
| **Operation time** | 308.97±48.192 | 340.77±32.191 | <0.001^*^ |
| **ICU** |  |  | 0.616 |
| No | 1373(92.8%) | 82(94.3%) |  |
| Yes | 106(7.2%) | 5(5.7%) |  |
| **Intraoperative infusion** | 3265.62±542.890 | 3313.22±537.677 | 0.795 |
| **Total in-hospital stay** | 20.84±6.693 | 21.30±4.251 | 0.529 |
| **Postoperative hospital stay** | 12.03±4.144 | 12.32±2.394 | 0.511 |
| **ICU stay** | 4.39±4.569(n=106) | 3.00±0.707(n=5) | 0.500 |

Supplementary Table 3 Information about the patient's surgery

Others*including Sweet esophagectomy, Ivor-Lewis esophagectomy, right thoracotomy with cervical anastomosis, mediastinoscopy-assisted esophagectomy; GEJ, gastroesophageal junction cancers

Supplementary Table 4 Postoperative complications in patients with esophageal cancer

| Characteristic | SEC  (N=1479) | COEC  (N=87) | P |
| --- | --- | --- | --- |
| **Clavien‒Dindo** |  |  | 0.713 |
| 1-2 | 664(44.9%) | 37(42.5%) |  |
| 3 | 235(15.9%) | 11(12.6%) |  |
| 4-5 | 53(3.6%) | 4(4.6%) |  |
| **Perioperative death** |  |  | 0.549 |
| No | 1457(98.5%) | 85(97.7%) |  |
| Yes | 22(1.5%) | 2(2.3%) |  |
| **Respiratory failure** |  |  | 0.788 |
| No | 1451 (98.1%) | 85(97.7%) |  |
| Yes | 28(1.9%) | 2(2.3%) |  |
| **Anastomotic stenosis** |  |  | 0.922 |
| No | 1381(93.4%) | 81(93.1%) |  |
| Yes | 98(6.6%) | 6(6.9%) |  |
| **Anastomotic leakage** |  |  | 0.464 |
| No | 1362(92.1%) | 82(94.3%) |  |
| Yes | 117(7.9%) | 5(5.7%) |  |
| **Pneumonia** |  |  | 0.598 |
| No | 1084(73.3%) | 66(75.9%) |  |
| Yes | 395(26.7%) | 21(24.1%) |  |
| **Atelectasis** |  |  | 0.311 |
| No | 1156 (78.2%) | 72(82.8%) |  |
| Yes | 323(21.8%) | 15(17.2%) |  |
| **Pulmonary consolidation** |  |  | 0.007^*^ |
| No | 1276 (86.3%) | 66(75.9%) |  |
| Yes | 203 (13.7%) | 21(24.1%) |  |
| **Pleural effusion** |  |  | 0.322 |
| No | 1232(83.3%) | 76(87.4%) |  |
| Yes | 247(16.7%) | 11(12.6%) |  |
| **Cardiac complication** |  |  | 0.930 |
| No | 1270(85.9%) | 75(86.2%) |  |
| Yes | 209 (14.1%) | 12(13.8%) |  |
| **DVT** |  |  | 0.212 |
| No | 1293(87.4%) | 80(92.0%) |  |
| Yes | 186(12.6%) | 7(8.0%) |  |

DVT, Deep vein thrombosis

Supplementary table 5 Clinicopathological characteristics of the subgroup of patients undergoing esophagectomy combined with lung resection after PSM

| Characteristic | SEC  (N=118) | | ECL  (N=59) | | P | | SMD |
| --- | --- | --- | --- | --- | --- | --- | --- |
| **Age** | 62.22±6.618 | | 62.86±7.033 | | 0.551 | | 0.094 |
| **Sex** |  | |  | | 0.893 | 0.021 | |
| Male | 95(80.5%) | | 48(81.4%) | |  | |  |
| Female | 23(19.5%) | | 11(18.6%) |  | | |  |
| **BMI** | 23.49±3.074 | | 23.87±2.791 | | 0.415 | | 0.132 |
| **Smoking** |  | |  | | 0.749 | | 0.051 |
| No | 65(55.1%) | | 31(52.5%) | |  | |  |
| Yes | 53(44.9%) | | 28(47.5%) | |  | |  |
| **Drinking** |  | |  | | 1.000 | | <0.001 |
| No | 74(62.7%) | | 37(62.7%) | |  | |  |
| Yes | 44(37.3%) | | 22(37.3%) | |  | |  |
| **Lung disease** |  | |  | | 0.684 | | 0.063 |
| No | 110(93.2%) | | 54(91.5%) | |  | |  |
| Yes | 8(6.8%) | | 5(8.5%) | |  | |  |
| **Diabetes** |  | |  | | 0.585 | | 0.084 |
| No | 114(96.6%) | | 56(94.9%) | |  | |  |
| Yes | 4(3.4%) | | 3(5.1%) | |  | |  |
| **Hypertension** |  | |  | | 1.000 | | <0.001 |
| No | 74(62.7%) | | 37(62.7%) | |  | |  |
| Yes | 44(37.3%) | | 22(37.3%) | |  | |  |
| **Coronary heart disease** |  |  | | | 1.000 | | <0.001 |
| No | 114(96.6%) | | 57(96.6%) | |  | |  |
| Yes | 4(3.4%) | | 2(3.4%) | |  | |  |
| **Surgical history** |  | |  | | 0.804 | | 0.039 |
| No | 90(76.3%) | | 44(74.6%) | |  | |  |
| Yes | 28(23.7%) | | 15(25.4) | |  | |  |
| **FVC** | 3.97±0.744 | | 3.64±0.721 | | 0.504 | | 0.122 |
| **FEV1** | 2.67±0.659 | | 2.68±0.629 | | 0.913 | | 0.018 |
| **DLCO** | 7.09±1.643 | | 7.20±1.567 | | 0.677 | | 0.067 |
| **EF** | 62.89±2.923 | | 62.86±2.063 | | 0.952 | | 0.010 |
| **ALB** | 40.20±3.633 | | 39.94±4.361 | | 0.676 | | 0.065 |
| **Neoadjuvant therapy** |  | |  | | 0.742 | | 0.052 |
| No | 73(61.9%) | | 38(64.4%) | |  | |  |
| Yes | 45(38.1%) | | 21(35.6%) | |  | |  |
| **Surgical procedures** |  | |  | | 0.560 | | 0.094 |
| McKeown | 98(83.1%) | | 51(86.4%) | |  | |  |
| Others* | 20(16.9%) | | 8(13.6%) | |  | |  |
| **Tumor location** |  | |  | | 0.239 | | 0.083 |
| Upper | 26(22.0%) | | 7(11.9%) | |  | |  |
| Middle | 33(28.0%) | | 23(39.0%) | |  | |  |
| Lower | 52(44.1%) | | 27(45.8%) | |  | |  |
| GEJ | 7(5.9%) | | 2(3.4%) | |  | |  |
| **Tumor size(cm)** | 3.24±1.400 | | 3.06±1.459 | | 0.433 | | 0.124 |
| **Histological type** |  | |  | | 0.884 | | <0.001 |
| Squamous | 105(89.0%) | | 53(89.8%) | |  | |  |
| Adenocarcinoma | 10(8.5%) | | 4(6.8%) | |  | |  |
| Other | 3(2.5%) | | 3(3.4%) | |  | |  |
| **TNM stage** |  | |  | | 0.824 | | 0.119 |
| 1 | 36(30.5%) | | 20(33.9%) | |  | |  |
| 2 | 42(35.6%0 | | 23(39.0%) | |  | |  |
| 3 | 36(30.5%) | | 14(23.7%) | |  | |  |
| 4 | 4(3.4%) | | 2(3.4%) | |  | |  |

BMI, body mass index; FVC, forced vital capacity; FEV1, forced expiratory volume in one second; DLCO, diffusing capacity of the lung for carbon monoxide; EF, [ejection](javascript:;) [fraction](javascript:;). Others*including Sweet esophagectomy, Ivor-Lewis esophagectomy, right thoracotomy with cervical anastomosis, mediastinoscopy-assisted esophagectomy; GEJ, gastroesophageal junction cancers
