# Supplementary figures and images for: Feasibility analysis of combined surgery for esophageal cancer
Source: World J Surg Oncol. 2023 Feb 10;21:41. doi: 10.1186/s12957-023-02930-0 (PMC9912580; doi:10.1186/s12957-023-02930-0)

Supplementary Figure 1. Patients with a previous surgical history and the top 10 surgical methods


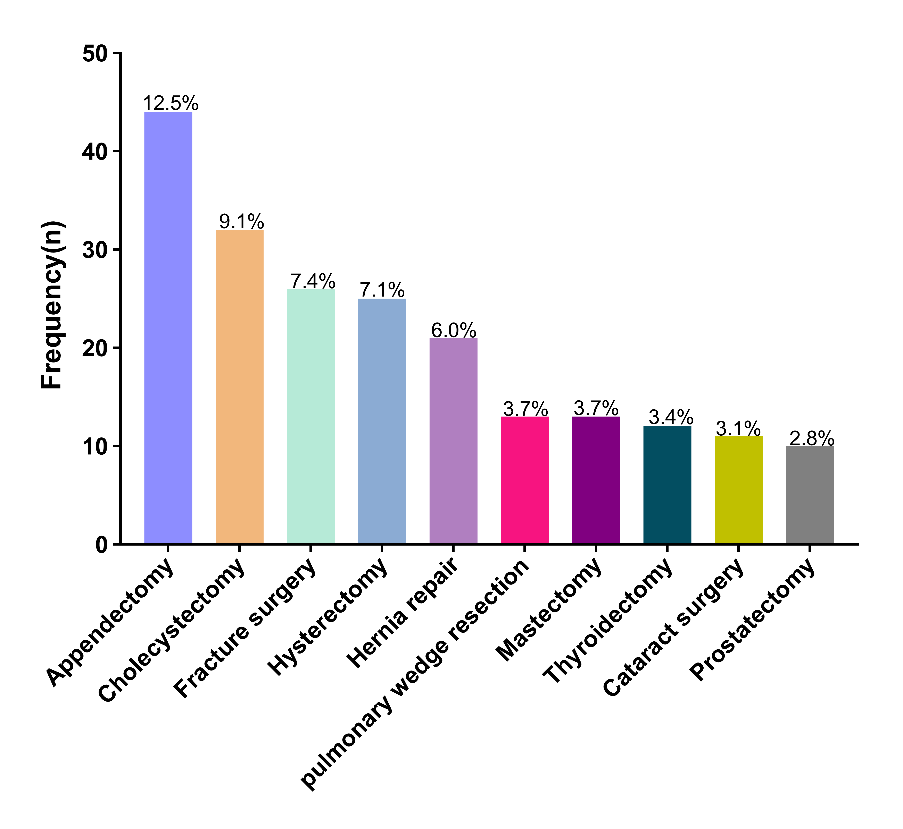

Supplement: Supplementary file 2 — Additional file 2: Supplementary Figure 1. Patients with previous surgery history, top 10 surgical method. [file 12957_2023_2930_MOESM2_ESM.docx]
